# Predictors of unmet health care needs in Serbia; Analysis based on EU-SILC data

**DOI:** 10.1371/journal.pone.0187866

**Published:** 2017-11-08

**Authors:** Natasa Popovic, Zorica Terzic-Supic, Snezana Simic, Biljana Mladenovic

**Affiliations:** 1 Institute of Social Medicine, Faculty of Medicine, University of Belgrade, Belgrade, Serbia; 2 Faculty of Economic, University of Belgrade, Belgrade, Serbia; Public Library of Science, FRANCE

## Abstract

Unmet health care needs have been designated as an indicator of equality in access to health care, which provides insight into specific barriers faced by respondents when they need medical services. The purpose of this research was to analyze demographic, socioeconomic, regional characteristics and perception of the health status; and identify predictors of unmet health care needs and consequently determine the size of inequalities in the availability, accessibility and acceptability of health care. The cross-sectional study obtained data from the Survey on Income and Living Conditions in the Republic of Serbia in 2014, based on a sample of 20,069 respondents over 16 years. Data was collected by using a household questionnaire and a questionnaire for individuals. Multivariate logistic regressions were applied. Almost every seventh citizen (14.9%) reported unmet health care needs. Predictors of unmet needs, for overall reasons, which increase the likelihood of their emergence included: self-perceived health status as very bad (OR = 6.37), divorced or widower/widow (OR = 1.31), living in the Sumadija region or Western Serbia (OR = 1.54) and belonging to the age group of 27 to 44 (OR = 1.55) or 45 to 64 years (OR = 1.52). The probability for those least reporting unmet health care needs included female patients (OR = 0.81), those with higher education (OR = 0.77), those who belong to the richest quintile (OR = 0.46) and who are unemployed (OR = 0.64). Reasons for unmet needs that indicate the responsibility of the health system amounted to 58.2% and reasons which represent preferences of the respondents amounted to 41.7%. The most frequent reason for unmet needs was financial (36.6%), and the wish to wait and see if the problem got better on its own (18.3%). Health policy should adopt a multidimensional approach and develop incentives for the appropriate use of health services and should eliminate barriers which restrict the accessibility and availability.

## Introduction

Socioeconomic inequalities are present not only in the population’s health status but also in access to and use of health care services [1, 2, 3]. Unequal access to basic health care services constitutes one of the social and economic determinants of health and therefore, is essential to identifying the scale of the problem for it to be addressed by the health system. Self-perceived unmet health care needs have been designated as a crucial indicator of equality of access to health care, which provides insight into specific barriers faced by respondents when they need medical services, and also represent an indicator of geographic, financial, cultural and physical accessibility of health care [4, 5]. Unmet health care needs are defined as the difference between the health services that are considered necessary for a particular health problem and services that are received [6]. Such needs represent an important indicator to measure inequalities in health and can be used as a complement to conventional methods in assessing the presence imbalances at the national and local levels [7, 8].

Access to health care is conditioned by the factors for which the health system is responsible and the factors that represent the individual preferences of people [4, 5, 9]. The responsibility of the health system is reflected in the availability and accessibility of efficient, high-quality, safe and affordable health services, which are cost-effective or justify the resources in their provision. The factors which represent the individual preferences of respondents and directly relate to the acceptability of health care are demographic and socioeconomic characteristics, previous experience of using health care, the perception of the benefits and quality of health services and the level of health literacy [4, 5, 7].

Availability is the degree to which appropriate health services are available to meet the needs of users, or in other words, the extent to which the health services provided to the scope, content and site, as part of the plan of the health care [10]. The organization of health services, the appointment system, the system of referral for specialist examinations, the quality of services provided and the way in which patients are treated within the health system also are factors in the accessibility to health services for which the health system is accountable [7]. The realization of the right to health care is reflected through waiting lists that have been established for diagnostic procedures and therapeutic interventions for which there is a greater need for the provision of the funds available. It is a response of the health system on the financial situation and ensures fairness in the delivery of health services that are working with limited resources [4, 5].

Accessibility represents the possibility of using health services and refers to the way that a person can get necessary medical services, by taking into account the physical (geographical distance, travel time), economic (cost of service, personal participation in the expenses, travel expenses, etc.) and social and cultural factors (language, ethnicity and religion) [10,11]. Acceptability relates to the extent to which health services are being used in practice, according to the norms and values of society [10]. Factors affecting the unmet health care needs which represent individual preferences of the respondents are the demographic and socioeconomic characteristics of the interviewees, their experience with health care, the perception of the benefits, the quality of health services and their level of health literacy [12].

Various approaches to measuring unmet needs are discussed based on clinical examinations or subjective assessment [6]. The clinical relies on a clinical assessment of whether an individual did not receive appropriate care [6]. The definition is based on clinical guidelines and, as a result, specific to a narrow set of conditions and treatments. Physicians have incomplete information about patients’ health care needs, and they rely on patients’ description of symptoms and history of illness, to make treatment decisions. Minority and lower socioeconomic groups may receive less effective health services because of the poor quality of communication among patients and physicians. One of reason could be the prejudice of doctors in the form of being less willing to cooperate with a patient from lower socioeconomic and minority groups, as well as clinical uncertainty associated with the differential interpretation of symptoms and stereotypes by doctors [13]. Consequently, these experiences may be perceived as unmet need by the patients.

The subjective assessment approach is more feasible due to various existing surveys including questions regarding unmet needs. Accordingly, in some way, subjective assessment of unmet health needs is superior to clinical assessment due to individuals being more able to estimate their health status as well as being in a unique position to identify their experiences and health needs [14]. Subjective assessment of unmet health needs may also include additional relevant information on the reasons and barriers for the unmet needs that can be employed to complement conventional methods of measuring and better understanding inequity which can be used to develop future health policy action [4].

The probability of experiencing unmet health care needs differs markedly between countries. This variability may be partially explained by differences in the financing and organization of the health system stated that universal coverage of health care, access rules and the free choice of general practitioners and access to specialist services without a referral, appointment system and the movement of patients are essential to the system to reduce the unmet health care needs [12]. In countries where the health system is organized in a way that patients are free to choose their general practitioner and visit a specialist without a referral, unmet health care needs are less (Belgium, Germany, Iceland, Luxembourg, Portugal) [12].

The greatest number of research related to unmet health care needs were carried out in the US and Canada, and in European countries such as Italy, Greece, Spain and Belgium [8, 15–19]. Even though a significant amount of research is applied to the representative sample of the population, there were also studies that have focused on specific population groups: poor people, the homeless, children with special health care needs, women during their reproductive period and elderly people [15, 20–27]. Some of them were cross-sectional or longitudinal studies that covered a large number of variables, analyzed as predictors of unmet medical needs in models of multivariate logistic regression (e.g. specific modalities of this technique, such as step-by-step regression, bivariate logistic regression), inequality index and relative concentration index. Several papers explored the issue of unmet health care needs using Horizontal inequity in health care use (using Horizontal Inequity Index), to measure the degree to which health care use is associated with income after controlling differences in need across the income distribution [28, 29, 30]. Horizontal equity is one of the wildly accepted concepts in health inequality research and demonstrates equal treatment of people in equal need regardless of sociodemographic factors such as income, sex, education and ethnicity [31, 32]. The Horizontal Inequity Index is equal to the difference between the income-related inequality in the unstandardized unmet need (unmet need concentration index) and the income-related inequality in need-expected care (need-expected concentration index). This approach also enables the decomposition of the contribution of need (i.e. self-assessed health) and non-need (i.e. socio-economic) variables to overall inequality in unmet health needs [33].

After 2010, the unmet health care needs in Europe had an average growth of 1.23% per annum [34]. The increase was attributed to an ageing population, the perception of the health, and its place in the value system, the rise of chronic noncommunicable diseases, scientific and technical innovation and the increase in health care costs. Unmet health care needs grow in health systems with universal coverage of the population; since the beginning of the global economic crisis in Europe, more than 1.5 million people have faced unmet health care needs [34].

### The health care system of the Republic of Serbia

Serbia inherited a health system from the former Yugoslavia that attempted to provide universal access and comprehensive health services for the population. Economic decline in Serbia, from the late 1980s, resulted in a substantial reduction in resources for health care in real terms. With the violent disintegration of the former Yugoslavia in 1991, the already weakened and structurally distorted economy of the RS of Serbia entered an acute phase with drastic consequences for the health care system and other social sectors.

The Serbian population of 7.1 million based on the model of current age structure, is one of the oldest in Europe and the world (average age 42.2 and 17.3 percent older than 65 years), characterized by smaller families and declining numbers of population in rural and remote areas [35]. The Health care system in Serbia is financed by compulsory health insurance contributions, based on 10.3% of payroll taxes [36]. The system formally provides access to comprehensive health services for the entire population. While public spending on health is relatively high (total health expenditures as a percentage of GDP is 10%), the total health expenditure per capita is among the lowest in the region (382 US$), due to low a GDP [36]. Mandatory health insurance covered 97% of the population in the field of preventive and other measures of health care [37]. Based on this data about 3% of the population is not insured, except in the area of emergency medical services. The recent changes in the laws with regard to The Law on Health Care and The Law on Health Insurance and the newly adopted legislation better regulate the rights of patients to health care and the wider coverage of health care insurance in the particular category of uninsured persons [38, 39].

In practice, the number of insured people is smaller due to the numerous problems faced by employees in companies undergoing restructuring and bankruptcy, so that the scope of health insurance is less than other populations. But no data on the exact number of such persons is available [35]. The government covers this through the transfer of funds for the health care of uninsured and vulnerable members of the population such as the elderly, the poor, refugees, displaced persons and Roma population albeit the amount for their health care needs is insufficient all these years. One in five inhabitants of the Republic of Serbia acquires the status of an insured person on this basis and funds for their health care are earmarked in the budget of the Republic of Serbia [35]. Since 2007, transfers from the budget for this purpose were significantly reduced to the extent that in 2014 these funds were 12.4 times smaller than those that were necessary to allocate according to the Law on Mandatory Social Insurance [40]. Regardless of the fact that the health care system in Serbia is based on the principles of accessibility (physical, geographical, economic and cultural) health care and the principle of equity, the differences are evident in health status, accessibility and use of health care services, the level of satisfaction with the services provided and out-of-pocket payments for the services received among vulnerable social groups and the majority population [41–43].

Health care for the Serbian population is provided through a well-developed network of 355 public health institutions organized at the primary, secondary and tertiary functional level [44]. Primary level care is delivered through a network of primary health care (PHC) centers. Public pharmacies are associated with PHC to dispense the prescriptions. Majority of hospitals are public (state-owned). Citizens with sufficient resources can access a burgeoning but largely unregulated private sector, concentrated mainly on outpatient and ambulatory care.

The Ministry of Health is the major decision maker in Serbian health care market. It develops health policies and budgets, monitors activities and approves plans for purchases of medical equipment. The whole system is highly centralized although some unsuccessful efforts were made to decentralized ownership of primary health care institutions. Decentralization is considered as an efficient way to improve the availability and better distribution of resources of health care and promotes community involvement in decisions about priorities in health care. Currently, the primary level of health care is mostly being established by local governments. The key obstacles in the way to effective decentralization are mainly the lack of financial resources in local government budgets, inherited economic and organizational problems, insufficient readiness for the acquisition of founding rights, as well as inadequate regulations in the field of health care. In some places, there are ineffective investments in health institutions, while in others, there are not enough resources to meet the demand for health care for the local population. Although various problems are related to a direct lack of resources that are difficult to overcome, there are also problems which are independent of this factor. Concerning the access to health care and the quality of the service provided, vulnerable groups, who are often discriminated against and socially excluded from these services, become unprotected and insecure. Working with limited financial resources in the health system of Serbia lead to the creation of longer waiting lists for specific and expensive medical procedures and interventions. In 2013, the number of new patients placed on waiting lists plus the length of waiting times for procedures or interventions significantly increased, which are considerably higher than other OECD's countries waiting lists [45]. Almost half of patients (46.6%) who were subjected to some intervention were placed on the waiting list, and only one-third of them have indeed received treatment [46]. The very weak flow of information about patients between health facilities and potential corruption is one of the major functional problems in waiting lists. The Serbian government has committed to improving and modernizing the national health system, and undertaken an extensive program of renovation, with the aid of external financial resources [47].

In the Republic of Serbia, there are no published papers about unmet health care needs, although there have been several national surveys which allowed an overview of the size of unmet health care needs which represent a generator of inequality in the health system. Results of a National Health Survey of the Republic of Serbia in 2013 showed that every third citizen faced unmet health care needs due to financial reasons, waiting lists for diagnostic and therapeutic procedures and distance from medical institutions [48]. The highest percentage of residents with unmet health care needs is registered in the region of Vojvodina (39.5%) and the lowest in the Sumadija region and Western Serbia (20.3%). The analysis of social demographic characteristics revealed that unmet health care needs were significantly higher in women (33.1%), among respondents with the lowest education (35.9%) and the poorest (40.1%).

According to the results of a Living Standard Measurement Survey in 2007, more than half of the diseased population believed that there was no need for the use of health care services 56%, and 26% thought that they could solve health problems by themselves. The main reason for non-use of health care services was the lack of financial resources (6%). Unmet health care needs were significantly higher in rural areas, and there were particularly pronounced differences between the respondents of the poorest and the richest quintile [49].

This research represents the first research on unmet health care needs in Serbia, conducted on a representative sample. The purpose of this research was to analyze the demographic, socioeconomic and regional characteristics of population with unmet health care needs, and their perception of their health status; to identify predictors of the unmet health care needs and consequently to determine the size of inequalities in the availability, accessibility and acceptability of health care and the responsibility of the health system of Serbia.

## Methods

### Sample design

This research was a cross-sectional study and represented a secondary analysis of data obtained from the Survey on Income and Living Conditions (SILC) in 2014, conducted on the territory of the Republic of Serbia, excluding Kosovo and Metohija [50, 51]. The principles of the Declaration of Helsinki were followed i.e. the result of the Survey are published on an aggregate level, and the anonymity of interviewed individuals and households is fully secured. Before interpretation of the result, the consent of the Ethical board from School of the Medicine University of Belgrade was obtained. The planned sample size was 8,008 households, in which were living 20,069 respondents. Respondents older than 16 years (16,219 respondents) have provided answers to questions about unmet health care needs and health status. The response rate was 80.8%. The sample consisted of all households enumerated in all the enumeration areas in the census of 2011, where the stratified rotating panel sample was used. The first stage units were the census circles and units of the second stage were households. Census circles as primary units were stratified according to the type of settlement to the densely populated, intermediate urbanized and thinly populated area and the four regions (Belgrade Region, Region of Vojvodina, Region of Sumadija and Western Serbia and the Region of Eastern and Southern Serbia).

### Questionnaires

Data was collected by using two questionnaires: a household questionnaire and a questionnaire for individuals. The household questionnaire contained 107 questions, including eight questions that referred to the gender, age, education, marital status and labor market status of household members, type of settlement and the region in which the household is located. The questionnaire for individuals contained 124 questions, including seven questions, which were related to the self-assessment of health status, the existence of chronic diseases, limitations in performing usual activities due to health reasons, visits to the doctor in the past 12 months and the main reasons why they did not visit a doctor.

### Variables

Self-perceived unmet health care needs of the population in the study were defined by the question: "Was there any time during the past 12 months that you should have visited a doctor, but did not?" Two groups of respondents were formed, those who had unmet health care needs and those with met medical needs. Respondents who responded that they had unmet health care needs were asked to answer to the main reason for not getting medical care. Answers that proposed were: could not afford to (too expensive), there is a waiting list, too far to travel to/distance from medical institution, could not take time because of work or care for children/others, fear of the doctor or hospital, wanted to wait and see if the problem got better on its own, did not know a good doctor or specialist and other reasons.

Potential predictors and independent variables of unmet health care needs are grouped by Andersen's Behavioral Model of Health Services Use, which assumes that the use of health services is a function of predisposing factors, factors that enable the use of health care and the factors that indicate the need to use health care [52]. Predisposing factors include socio-demographic variables (age, sex, level of education, marital status and work activity). Gender is encoded as male and as female, and age is categorized into age groups: 16–26, 27–44, 45–64 and 65+ years. Education is encoded as primary, secondary and tertiary education, and marital status as married/unmarried, divorced / widowed. Factors that enable utilization of health care services represented the personal, family and social resources that may enable or represent a barrier to accessing health care and was presented as equivalent household disposable income. The equalized household disposable income is the total disposable household income modified according to the OECD scale of equivalence in the structure of the household, which were divided into five quintiles. Total disposable household income includes all of the net monetary income of each household member and all income at the household level, during the reference year (such as work employee wages and self-employment earnings, income from investment and property, social benefits and pensions, which are reduced by taxes paid and contributions. Social transfers are not taken into account. Factors that indicate the need for health services were presented with variables: the self-perceived health status of the population (five-point scale from very good to very bad), the presence of chronic conditions and the presence of limitations in daily activities due to health problems.

Following previous literature on reasons for unmet health care needs, we have divided them into three categories, according to the nature of the stated reason [17, 18, 25, 53]. The first group of responses, which threatened the availability of the health care, was waiting lists for interventions at the time required. The second group of reasons threatened the accessibility of health care and relates to barriers such as financial challenges and distance from medical institutions. The third category of responses has threatened the acceptability of health care related to the personal circumstances of responders, like fear of doctors or treatment, wanted to wait and see if the problem got better on its own, did not know any good doctor or specialist and other reasons.

### Statistical analysis

Data were expressed as the absolute number and frequency (percentages) and Pearson's chi-square test was used to analyze the differences among of respondents with unmet health care needs and those who have met health care needs for weighted values. Multicollinearity was analyzed using the Variance Inflation Factor (VIF). All variables have VIF less than three which is standard cut-off value for multicollinearity. Almost, all variables have VIF less than 2, except age VIF 2.2 and general health VIF 2.7. As both variables should be in model and VIF is below a usual threshold, we assume that no multicollinearity is identified in the multivariate model.

Variables which were significant in the univariate models of logistic regression were used as the independent variables in the multivariate logistic regression models. Nine logistic regressions models were performed. In the first logistic regression model, the dependent variable was unmet health care needs for all reasons. The second logistic regression model for the dependent variable had unmet health care needs due to reasons that indicate the threatened availability of health care. The dependent variable in the third model were unmet health care needs for reasons that show the threatened accessibility and the fourth logistic regression model the dependent variable was unmet health care needs that threatened the acceptability of health care. The responsibility of the health system for unmet health care needs reflecting in the reasons that relate to the threatened availability and accessibility of health care services presented the dependent variable in the fifth model of logistic regression. From the sixth to ninth logistic regression models the dependent variables were unmet medical needs according to four regions; Belgrade Region, Region of Vojvodina, Region of Sumadija and Western Serbia, and Region of Eastern and Southern Serbia. The odds ratios (OR) and 95% confidence intervals (CIs) were calculated. The results were considered statistically significant when the p-value was ≤ 0.05. The analysis was performed using the Statistical Package for Social Sciences (SPSS) version 20.

## Results

Almost 14.9% of the Serbian population, aged 16 and over, reported they had unmet health care needs for medical examinations or treatment. The social-demographic characteristics of the population reporting unmet and met medical needs are presented in Table 1. There was a statistically significant difference between respondents with met and unmet health care needs. Male respondents more often reported unmet health care needs. The highest percentage of respondents with unmet needs was in the age group of 45–64 years, or in the group of the older active working population.

**Table 1:** Social-demographic characteristics of the population reporting unmet and met health care needs.

Variables	Population reporting unmet health care needs	Population reporting met health care needs	p-value*
	N = 2,389 (14.9%)	N = 13,830 (85.1%)	
***Predisposing factors***			
**Sex**:			<0.001
Male	1,187 (15.2)	6,628 (84.8)	
Female	1,202 (14.5)	7,202 (85.5)	
**Age**:			<0.001
16–26	110 (5.1)	2,207 (94.9)	
27–44	534 (12.5)	3,834 (87.5)	
45–64	1,150 (20.3)	4,618 (79.7)	
65+	549 (15.5)	3,171 (84.5)	
**Education**:			<0.001
Primary education	967 (18.4)	4,453 (81.6)	
Secondary education	1,163 (14.2)	7,190 (85.8)	
Tertiary education	259 (10.6)	2,187 (89.4)	
**Employment status**:			<0.001
Employment	902 (15.8)	4,817 (84.2)	
Unemployment	518 (15.9)	2,820 (84.1)	
In pension	687(15.4)	3,818 (84.6)	
Inactive	229 (9.0)	2,148(91.0)	
**Marital status**:			<0.001
Unmarried	382 (9.4)	3,816 (90.6)	
Married	1,407 (15.6)	7,719 (84.4)	
Widowed	188 (19.9)	654 (80.1)	
Divorced	412 (21.5)	1,641 (78.5)	
***Enabling factors***			
**Equalized disposable income quintile**:			<0.001
0–20	713 (21.6)	2,551 (78.4)	
20–40	529 (15.7)	2,719 (83.4)	
40–60	430 (13.4)	2,815 (86.6)	
60–80	410 (12.5)	2,811 (87.5)	
80–100	307 (10.2)	2,934 (89.8)	
***Need factors***			
**Health status**:			<0.001
Very good	171 (4.5)	3,891 (95.8)	
Good	626 (12.7)	4,470 (87.3)	
Fair	857 (22.9)	3,030 (77.1)	
Bad	622 (22.9)	2,048 (77.1)	
Very bad	113 (21.0)	391 (79.0)	
**Suffering from any chronic condition**:			<0.001
Chronic	1,038 (20.9)	3,820 (79.1)	
No chronic condition	1,351 (12.3)	10,010 (87.7)	
**Limitation in daily activities**:			<0.001
Quite limited	174 (21.7)	637 (78.3)	
Limited	432 (22.5)	1,477 (77.5)	
Not limited	1,783 (13.4)	11,716 (86.6)	
**Permanent disability**:			<0.001
No	2,171 (14.6)	12,816 (85.4)	
Yes	218 (18.1)	1,014 (81.9)	
***Geographical area***			
**Region**:			<0.001
Belgrade region	305 (11.7)	2,357 (88.3)	
Region of Vojvodina	841 (19.3)	3,623(80.7)	
Region of Sumadija and Western Serbia	718 (13.9)	4,521 (86.1)	
Region of East and South Serbia	525 (14.0)	3,329 (86.0)	
**Degree of urbanization**:			<0.001
Densely populated area	690 (13.5)	4,336 (86.5)	
Intermediate urbanized area	646 (14.9)	3,893 (85.1)	
Thinly populated area	1,053 (16.2)	5,601 (83.8)	

*p-value: Chi-square test on the difference of unmet and met needs of health care across different socio-demographic groups.

With increasing educational level, the frequency of unmet health care needs for health services are reduced, and they are the largest in the group of respondents with elementary education. According to employment status, unemployed, employed and retired respondents were not much different in the frequency of unmet health care needs (from 15.9% to 15.4%).

Unmet health care needs were greatest in the group of divorced and widowed respondents while married respondents had a bit higher unmet needs than the national average. The largest percent of respondents with unmet health care needs were living in households belonging to the first poorest quintile, and with the increased income of the household, unmet health care needs decrease. In the richest quintile, the number of those with unmet health care needs is two times lower than the first poorest quintile (Table 1).

Unmet health care needs were most frequently in the category of respondents who have assessed their health as very bad and with the deteriorating health condition of respondents are growing unmet health care needs. The existence of chronic diseases significantly affected the increasing frequency of unmet health care needs. The presence of long-standing limitations due to health problems and permanent disability contributes to increasing unmet health care needs (Table 1).

Regional variations in the frequency of unmet health care needs were significant: the Belgrade region had the lowest frequency of unmet health care needs (11.7%), while the region of Vojvodina had the highest frequency (19.3%). Analysis of the degree of urbanization revealed the existence of the highest frequency of unmet health care needs in thinly populated areas, while in densely populated areas the frequency of unmet needs were significantly below the average of Serbia (Table 1).

Reasons for unmet health care needs that indicate the responsibility of the health care system (threatening the accessibility and availability of health care) amounted to 58.2% (Table 2). The most frequent reason in the context of the accessibility of health care in all regions was financial reasons (36.6%), which has been particularly noticeable in the region of Vojvodina (42.4%) and somewhat less in the region of Eastern and Southern Serbia (41.8%). In the Belgrade area, financial reasons were the least represented cause of unmet needs.

**Table 2:** The main reason why respondent did not visit a doctor.

Reason why respondent did not visit a doctor	N	%
**Responsibility of health care system**	1,186	58.2
Accessibility	847	40.5
Could not afford it/too expensive	770	36.6
It is too far to travel	77	3.9
Availability	339	17.7
There is a waiting list	339	17.7
**Reasons related to the personal circumstance of responders**	859	41.7
Acceptability	859	41.7
Could not find the time because of work, care of children or others	306	16.1
Fear of doctors/hospital/testing/treatment	120	5.7
Wanted to wait and see if the situation was going to get better	402	18.3
Did not know of any good doctor or specialist	31	1.6
For other reasons	0	0

Distance from health centers was one of the least represented reasons for unmet needs in all regions, from 2% in the Belgrade region to 56% in the region of Sumadija and Western Serbia. Within threatened accessibility, waiting lists for medical interventions and procedures were the highest in the Belgrade region (27.8%) and the lowest in the region of Sumadija and Western Serbia (12.5%) (Table 2).

**Fig 1:**
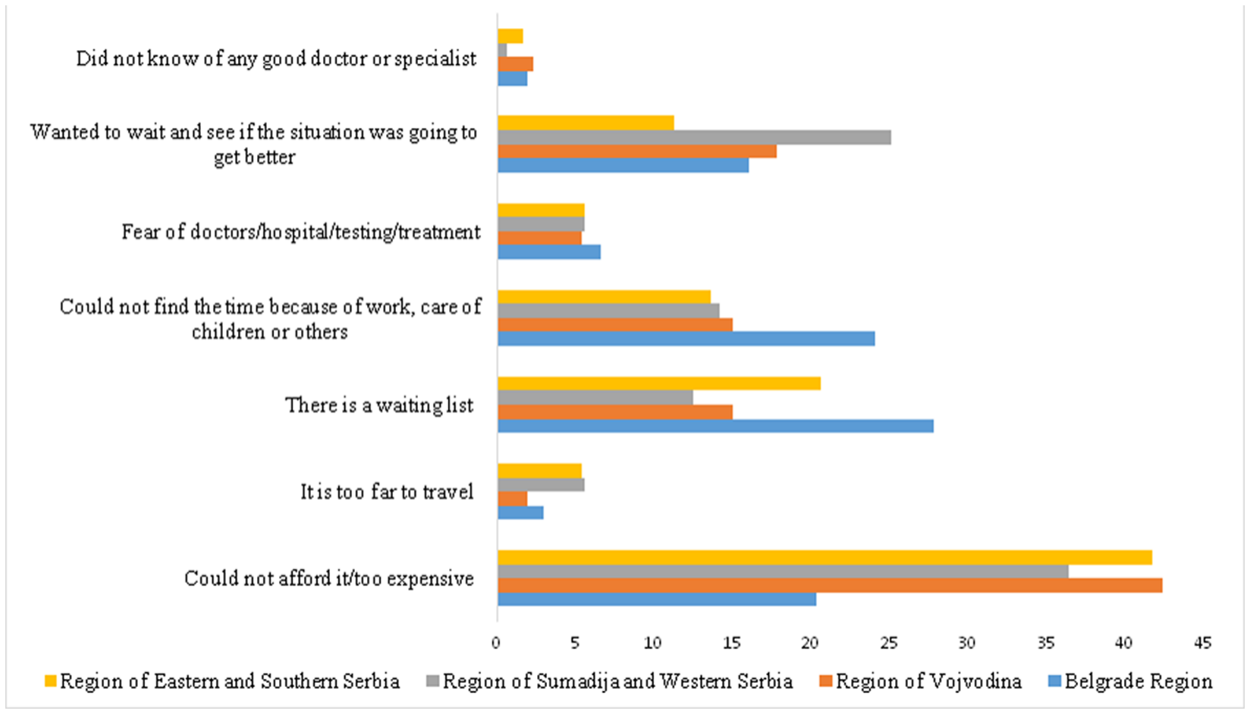
The main reason why respondent did not visit a doctor, by regions.

Reasons that related to the individual preferences of the respondents or threatened the acceptability of health care represented 41.7% of all reasons. The most frequent reasons in the context of acceptability were the wish to wait and see if the problem got better on its own, and the lack of time due to employment or childcare. In the Belgrade region, which is the most densely populated area, the lack of time due to work and care for children had the highest frequency (24.1%) (Fig 1). In the region of Sumadija and Western Serbia, the most frequent reason was waiting to see if the problem got better on its own (25.1%) (Fig 1).

The five models of the multivariate logistic regression used to show the association of demographic, socioeconomic and regional characteristic of the population and unmet health care needs (Table 3).

**Table 3:** Multivariate logistic regression models for five categories of reasons unmet health care needs.

Variables	The categories of the reason unmet health care needs, 2014				
	Model 1 Overall OR (95% CI)	Model 2 Availability OR (95% CI)	Model 3 Accessibility OR (95% CI)	Model 4 Acceptability OR (95% CI)	Model 5 Responsibility of the health care system OR (95%CI)
**Sex**					
Male	1.0	1.0	1.0	1.0	1.0
Female	0.81 (0.74–0.90)*	0.99 (0.79–1.25)	0.89 (0.76–1.04)	0.80 (0.69–0.93)*	0.91 (0.8–1.04)
**Age**					
16–26	1.0	1.0	1.0	1.0	1.0
27–44	1.55 (1.22–1.97)*	1.84 (0.93–3.78)	1.62 (1.06–2.48)*	1.37 (0.95–1.98)	1.72 (1.20–2.48)*
45–64	1.52 (1.16–1.94)*	2.32 (1.12–4.80)*	1.42 (0.90–2.21)	1.25 (0.84–1.84)	1.7 (1.20–2.48)*
65+	0.86 (0.65–1.10)	2.04 (0.95–4.38)	0.59 (0.37–0.95)*	0.81 (0.52–1.25)	0.86 (0.57–1.29)
**Education**					
Primary education	1.0	1.0	1.0	1.0	1.0
Secondary education	0.92 (0.82–1.02)	1.37 (1.03–1.82)	0.61 (0.52–0.73)*	1.19 (0.99–1.42)	0.75 (0.65–087)*
Tertiary education	0.77 (0.65–0.92)*	1.33 (0.90–1.95)*	0.38 (0.27–0.56)*	1.05 (0.82–1.36)	0.62 (0.48–0.80)*
**Employment status**					
Employment	1.0	1.0	1.0	1.0	1.0
Unemployment, in pension and inactive	0.64 (0.58–0.72)*	0.78 (0.59–1.03)	0.79 (0.66–0.95)*	0.60 (0.51–0.71)*	0.78 (0.67–0.91)*
**Marital status**					
Married	1.0	1.0	1.0	1.0	1.0
Unmarried	0.95 (0.82–1.11)	1.05 (0.72–1.52)	1.03 (0.81–1.31)	0.73 (0.58–0.92)*	1.04 (0.85–1.27)
Divorced/Widowed	1.31 (1.15–1.48)*	1.30 (0.98–1.73)	1.21 (1.00–1.46)	1.11 (0.91–1.35)	1.23 (1.04–1.45)*
**Equalized disposable income quintile**					
0–20%	1.0	1.0	1.0	1.0	1.0
20–40%	0.73 (0.64–0.84)*	1.07 (0.77–1.49)	0.54 (0.45–0.65)*	1.16 (0.92–1.45)	0.61 (0.52–0.73)*
40–60%	0.59 (0.51–0.68)*	0.90 (0.63–1.28)	0.35 (0.28–0.44)*	1.10 (0.87–1.40)	0.43 (0.35–0.52)*
60–80%	0.59 (0.51–0.69)*	0.78 (0.54–1.14)	0.40 (0.32–0.51)*	1.17 (0.92–1.48)	0.45 (0.37–0.54)*
80–100%	0.46 (0.39–0.54)*	0.90 (0.62–1.31)	0.09 (0.06–0.14) *	0.95 (0.74–1.22)	0.25 (0.19–0.32)*
**Health status**					
Very good	1.0	1.0	1.0	1.0	1.0
Good	2.77 (2.30–3.34)*	2.21 (1.37–3.55)*	2.1 (1.46–3.02)*	2.81 (2.16–3.67)*	2.19 (1.64–2.92)*
Fair	5.82 (4.76–7.13)*	3.71(2.23–6.167)*	6.51 (4.50–9.44)*	4.56 (3.40–6.11)*	5.71 (4.22–7.71)*
Bad	6.34 (5,0–8.05)*	5.0 (2.79–8.95)*	8.85 (5.86–370)*	3.65 (2.53–5.27)*	7.80 (5.56–10.96)*
Very bad	6.37 (4.58–8.85)*	2.38 (0.98–5.76)	11.06 (6.67–8.33)*	2.21(1.12–4.36)	7.80 (5.06–12.03)*
**Suffering from any chronic condition**					
No chronic condition	1.0	1.0	1.0	1.0	1.0
Chronic	1.12 (0.99–1.27)	1.01 (0.76–1.34)	1.08 (0.90–1.30)	1.14 (0.95–1.37)	1.07 (0.91–1.25)
**Limitation in daily activities**					
Not limited	1.0	1.0	1.0	1.0	1.0
Limited	1.04 (0.91–1.19)	1.30 (0.95–1.76)	1.03 (0.84–1.25)	0.75 (0.59–0.95)*	1.11 (0.93–1.31)
**Degree of urbanization**					
Densely populated area	1.0	1.0	1.0	1.0	1.0
Intermediate urbanized area	0.86 (0.76–0.98)*	0.75 (0.56–0.99)*	1.10 (0.87–1.38)	0.83 (0.69–1.00)	0.92 (0.77–1.10)
Thinly populated area	0.91 (0.81–1.03)	0.55 (0.41–0.73)*	1.41 (1.15–1.73)*	0.81 (0.68–0.98)	1.03 (0.87–1.22)
**Region**					
Belgrade Region	1.0	1.0	1.0	1.0	1.0
Region of Vojvodina	0.99 (0.84–1.17)	0.98 (0.69–1.39)	0.74 (0.54–1.01)	1.17 (0.90–1.52)	0.91 (0.72–1.14)
Region of Sumadija and Western Serbia	1.54 (1.36–1.75)*	0.96 (0.71–1.29)	1.61 (1.32–1.96)*	1.70 (1.38–2.10)*	1.40 (1.19–1.66)*
Region of Eastern and Southern Serbia	1.0 (0.88–1.13)	0.70 (0.51–0.95)*	0.99 (0.81–1.21)	1.53 (1.24–1.89)*	0.90 (0.76–1.07)

OR—Odds ratio; CI—Confidence interval.

* Significant

Our result showed that women statistically reported less unmet health care needs due to all reasons (OR = 0.81) and acceptability (OR = 0.80) (Model 1 and 3). The probability of experiencing unmet health care needs are reduced with increasing age in all models except due to threatened availability, where the likelihood of unmet health care needs is the highest in the age group from 45–64 (OR = 2.32). Having a higher level of education and being unemployed, in pension or inactive significantly reduced the likelihood for unmet health care needs. Widowed and divorced respondents were significantly more likely to report unmet health care needs due to overall reason (OR = 1.31) and due to the responsibility of health care system (OR = 1.23). Being unmarried reduced the likelihood for unmet health care needs due to acceptability reason. (OR = 0.73) (Table 3).

Increasing income significantly reduces the likelihood of unmet health care needs, and it is the smallest in the richest quintile. The health status showed a strong statistically significant association with unmet health care needs so that the deterioration of health conditions increases the probability of unmet health care needs in all models (OR = 11.06, Model 3). The presence of long-standing limitations due to health problems and permanent disability contributes to decreasing unmet health care needs only due to threatened acceptability. (OR = 0.75). Having any chronic condition is not associated with the probability of unmet health care needs (Table 3).

In thinly populated areas the probability of experiencing unmet health care needs are reduced due to threatened availability (OR = 0.55), while in the same areas respondents had the greatest likelihood to have unmet health care needs due to the threatened accessibility (OR = 1.41). Respondents in intermediate urbanized areas were less likely to report unmet health care needs due to overall reason (OR = 0.86) (Table 3).

Regional inequality in reasons for unmet needs was significantly present. The highest probability of unmet health care needs due to all reason (OR = 1.54), accessibility (OR = 1.61), acceptability (OR = 1.70) and responsibility of the health care system (OR = 1.40) was in the region of Sumadija and Western Serbia. At the same time in the region of Eastern and Southern Serbia, the likelihood of experiencing unmet health care needs due to threatened availability was significantly lower (OR = 0.70), while threatened acceptability increases the probability of experiencing unmet health care needs (OR = 1.53) (Table 3).

Differences in the odds of unmet health care needs were observed among geographical areas in Table 4. In the Region of Sumadija and Western Serbia, as well as Eastern and Southern Serbia women reported less unmet health care needs.

**Table 4:** Multivariate logistic regression models of overall reasons of unmet health care needs by regions.

Variables	Regions			
	Belgrade region OR (95% CI)	Region of Vojvodina OR (95% CI)	Region of Sumadija and Western Serbia OR (95% CI)	Region of Eastern and Southern Serbia OR (95% CI)
**Sex**				
Male	1.0	1.0	1.0	1.0
Female	0.92 (0.70–1.19)	0.85 (0.72–1.01)	0.77 (0.64–0.92)*	0.76 (0.62–0.93)*
**Age**				
16–26	1.0	1.0	1.0	1.0
27–44	3.04 (1.31–7.07)*	1.41 (0.97–2.06)	1.34 (0.82–2.20)	1.49 (0.88–2.51)
45–64	2.48 (1.03–5.99)*	1.33 (0.89–1.98)	1.35 (0.80–2.28)	1.76 (1.02–3.03)*
65+	1.16 (0.45–2.96)	0.67 (0.43–1.05)	0.89 (0.51–1.55)	1.09 (0.61–1.96)
**Education**				
Primary education	1.0	1.0	1.0	1.0
Secondary education	1.03 (0.71–1.51)	0.95 (0.79–1.15)	0.91 (0.75–1.12)	0.89 (0.71–1.13)
Tertiary education	1.01 (0.64–1.59)	0.78 (0.58–1.05)	0.68 (0.48–0.96)*	0.76 (0.52–1.11)
**Employment status**				
Employment	1.0	1.0	1.0	1.0
Unemployment. in pension and inactive	0.67 (0.49–0.91)*	0.74 (0.61–0.90)*	0.52 (0.43–0.63)*	0.74 (0.58–0.95)*
**Marital status**				
Married	1.0	1.0	1.0	1.0
Unmarried	0.65 (0.43–0.99)*	1.00 (0.79–1.28)	0.93 (0.70–1.25)	1.07 (0.77–1.49)
Divorced/Widowed	1.26 (0.90–1.77)	1.13 (0.90–1.41)	1.52 (1.22–1.91)*	1.33 (1.03–1.72)*
**Equalized disposable income quintile**				
0–20%	1.0	1.0	1.0	1.0
20–40%	0.80 (0.49–1.31)	0.81 (0.64–1.02)	0.78 (0.61–0.99)*	0.62 (0.47–0.80)*
40–60%	1.19 (0.75–1.88)	0.53 (0.41–0.68)*	0.69 (0.54–0.89)*	0.44 (0.32–0.60)*
60–80%	1.05 (0.65–1.66)	0.58 (0.45–0.74)*	0.55 (0.42–0.72)*	0.59 (0.42–0.82)*
80–100%	0.87 (0.55–1.37)	0.42 (0.32–0.56)*	0.41 (0.29–0.57) *	0.40 (0.28–0.55)*
**Health status**				
Very good	1.0	1.0	1.0	1.0
Good	2.44 (1.59–3.77)*	2.26 (1.66–3.08)*	3.12 (2.14–4.55) *	3.69 (2.41–5.66)*
Fair	5.92 (3.61–9.70)*	4.75 (3.40–6.65)*	6.88 (4.59–10.32)*	6.51 (4.12–10.31)*
Bad	4.35 (2.29–8.26)*	6.20 (4.15–9.28)*	7.12 (4.50–11.26)*	6.70 (3.93–11.41)*
Very bad	3.43 (1.12–10.55)*	3.95 (2.17–7.23)*	12.97 (7.35–22.89)*	4.82 (2.32–10.04)*
**Suffering from any chronic condition**				
No chronic condition	1.0	1.0	1.0	1.0
Chronic	0.94 (0.67–1.33)	1.27 (1.03–1.56)*	1.24 (0.10–1.55)	0.89 (0.69–1.16)
**Limitation in daily activities**				
Not limited	1.0	1.0	1.0	1.0
Limited	1.02 (0.67–1.55)	1.12 (0.89–1.41)	0.89 (0.69–1.14)	1.18 (0.88–1.57)
**Degree of urbanization**				
Densely populated area	1.0	1.0	1.0	1.0
Intermediate urbanized area	0.65 (0.44–0.96)*	0.88 (0.71–1.08)	0.63 (0.47–0.83)*	1.24 (0.95–1.64)
Thinly populated area	0.89 (0.64–1.25)	0.89 (0.71–1.11)	0.88 (0.71–1.09)	0.99 (0.75–1.29)

OR—Odds ratio; CI—Confidence interval.

* Significant

In the Belgrade Region and the Region of Eastern and Southern Serbia, the probability of experiencing unmet health care needs was reduced with increasing age; it was the highest among the working population. The level of education did not appear to be significantly associated with unmet needs except in Region of Sumadija and Western Serbia, where a higher level of education reduced the probability of having unmet health care needs. Economically inactive people were less likely to experience an unmet health care need in all regions.

The greatest likelihood of unmet health care needs had divorced/widowed respondents in the Region of Sumadija and Eastern and, Southern Serbia. An increased income decreased the probability of unmet health care needs in all regions, except in the Belgrade Region. The health status showed an association with unmet health care needs in all regions, in particular among respondents with a very bad health in the Region of Sumadija and Western Serbia (OR = 12.97). The presence of chronic condition was associated with the probability of unmet heath care needs only in the Region of Vojvodina. Living in an intermediate urbanized area of the Belgrade Region and Region of Sumadija and Western Serbia decreased the probability of experiencing unmet health care needs.

## Discussions

Our analysis indicated that almost every seventh citizen of Serbia (14.9%) had unmet health care needs which are much higher in comparison to the other 28 European countries that have conducted SILC surveys, where the average unmet health care needs were 6.9% [9]. In comparison to the countries of the former Yugoslavia, Serbian respondents had the highest frequency of unmet health care needs (Montenegro 12.7%, Macedonia 10.8%, Croatia 7.5% and Slovenia 0.4%) [9].

The responsibility of the health system in Serbia, which is reflected in the availability and accessibility of health care, was higher than the responsibilities of the respondents or the acceptability of health care. Concerning the responsibility of the health care system, which represents availability and accessibility, 28 countries of Europe were almost at the same level as in Serbia, and the order of the reasons for unmet health care needs has shown a nearly identical pattern [9]. The most common reason for unmet health care needs for accessibility was financial; waiting lists for diagnostic procedures were most common with regard to availability [9]. We found that the most dominant reason in the context of threatened acceptability was waiting to see if the problem got better on its own, while in the 28 European countries it was the lack of time due to work and care for their children [9]. Our results indicate that demographic and socioeconomic characteristics as well as the health status of respondents have a significant role in explaining unmet health care needs. Women are less likely to report unmet health care needs for overall reasons as well as due to the acceptability of health care, which was opposite to other studies, where women reported higher levels of unmet needs for health care [18, 28, 54, 55]. A possible explanation is the fact that women are more likely to visit a doctor due to a higher level of awareness about the health needs, problems and symptoms of the disease compared to men, that was confirmed by the Results of National Health Survey of Republic of Serbia in 2013, where women (71.6%) have significantly more frequently visited a general practitioner compared to men (59%) [48]. In a study conducted in Italy and Greece, the likelihood of experiencing unmet health care needs due to reasons of overall accessibility and acceptability were higher among women [17, 18]. Our findings showed that the probability of experiencing unmet health care needs due to the overall responsibility of the health care system was reduced with an increase in age, with the only exception being the availability of health care. Respondents in the age group of 45–64 years were twice as likely to report unmet health care needs due to availability, which was contradictory to other findings, where being young was positively associated with unmet needs [18, 55, 56]. We highlighted that one of the possible explanations for a higher frequency of older employees with unmet health care needs is the inability to exercise the right to health care due to unpaid taxes and contributions for health insurance as a consequence of companies undergoing restructuring and liquidation; although these workers were formally entitled to rights related to health care, they face practical obstacles in exercising them [35].

Our study has identified educational disparities in unmet health care needs; except for availability, having a higher level of education reduced the likelihood of unmet health care needs. This is consistent with a previous study in Serbia, which found that people with higher levels of education have a higher social status, more stable and larger income, use more private health services and pay more out-of-pocket for needed health services [41, 57]. Furthermore, the general pattern of reducing unmet health care needs with an increase in education levels in Serbia is consistent with countries that have the largest share of a population with secondary education, such as Bulgaria and Croatia [9].

Unemployment has detrimental effects on the health of individuals and their families, which carry psychological, physical and financial consequences [58]. In line with previous studies, we find that the probability of experiencing unmet health care needs in each model is significantly lower for unemployed respondents [18, 55]. This connection could be explained by the fact that working individuals experienced more difficulties in being able to take time off to seek health care. Likewise, employees of companies undergoing restructuring and bankruptcy faced various problems in exercising their rights to health care, due to unregulated obligations for medical insurance contributions.

Analyzing the relationships between professionally defined needs, use of health services and unmet health care needs, the same research has shown that divorced people have more contact with professional health care providers (general practitioners, psychiatrists, psychologists), due to social and emotional problems [24]. Our results were consistent with the results of another study, where the likelihood of unmet health care needs were largest in the group of divorced respondents [18], due to overall reasons and the responsibility of the health care system. The protective factor that reduces the probability of the existence of unmet health care needs due to the acceptability is to be unmarried.

The enabling factor showed a strong association with unmet health care needs. Consistent with previous research, our study showed that increasing equalized disposable income decreased the likelihood of unmet health care needs in all models, but it was statistically significant in the models that indicate responsibility of the health care system, accessibility and overall reasons for unmet needs [18, 53, 59–63]. On the contrary, studies from countries with universal health care coverage report only slight associations between unmet health care and income [55, 60, 62, 63]. Increasing coverage of health insurance and the formulation of specific health policies may reduce inequalities in access to health care, particularly for vulnerable population groups [64].

Our results confirmed the claims from the previous studies that the deterioration of the state of health and the existence of chronic diseases and functional restrictions lead to an increase in unsatisfied health needs, which has been particularly noticeable in the Region of Sumadija and Western Serbia [18, 59]. On the other hand, barriers to accessing health care have negative consequences for the health of poor people with chronic diseases, conditions and functional limitations in performing daily activities [18, 55, 60, 63]. In our research, health status indicates a strong association with unmet needs so that the deterioration of health conditions increases the probability of unmet needs in all multivariate modes except with regard to availability and acceptability in the category of respondents who assessed their health as very bad. Respondents who assessed their health as very bad had a greater likelihood to have unmet needs for all reasons as well as accessibility. A possible explanation may be the adoption of the rules on waiting lists in 2013, which defines the methodology for the formation of waiting lists, along with the criteria and standardized measures for assessing the health status of an insured person [45]. Patients with chronic illnesses have more often reported unmet health care needs, and they have grown with the increasing number of chronic diseases [25]. Unexpectedly, in our study the presence of chronic conditions was not statistically significantly associated with unmet health care needs in all categories of reasons.

Regional inequality of access to basic health services is significantly present in Serbia, despite systemic laws and the adopted principles of physical, geographical, economic and cultural accessibility of health care and the principle of equity. Surprisingly, the region of Eastern and Southern Serbia, which has the highest percentage of respondents in the poorest quintile of income, does not have the highest frequency of unmet health care needs. At the same time, the region of Vojvodina had the highest frequency of unmet health care needs particularly in thinly populated areas, among respondents who belong to the poorest quintile of income. One of the reasons could be that in thinly populated areas satellite clinics and outpatient facility in rural areas had been closed due to cost-saving reforms in the health system during the economic crisis, which resulted in a lower accessibility of these services to the rural, predominantly elderly population [35]. It is important to highlight that in thinly populated regions we have a higher number of elderly populations, which usually live in households that subsist on agriculture. Agricultural households are significantly more likely to face problems related to the mandatory health insurance due to the unregulated formal status of farmers and cadastral taxes, which present a substantial basis for the regulation of the official status of social rights. Cuts to health care services, closure of facilities, reduced opening hours and numbers of health care personnel contributed to worsening access to health care [34].

The strength of this study is that it represents the first research, based on a large nationally representative sample, on unmet health care needs in Serbia under conditions of the growing vulnerability of certain population groups in the health system, which generates inequalities in health. The implementation of continuous research using the same methodology with the same respondents allows us to perform longitudinal studies and enables comparison with other countries which conducted the SILC survey. Research on unmet health care needs can contribute to consideration of the responsibility of the health care system and the responsibility of respondents, and suggest measures and health policies that would contribute to reducing health inequalities and improving health for vulnerable population groups as well as the whole population.

This study has some limitations. All data on unmet health care needs is based on self-perception and therefore, to some extent, reflects the subjective experience that is influenced by the social and cultural environment of the participants of the SILC. Data obtained in this study did not include those who were living in institutions of health and social care (hospitalized or in nursing homes) and whose state of health is much more severe than those living in their homes. That is why information about the state of health, in general, can be underestimated. On the other hand, exclusion of these persons, which for the health service is always available, can lead to overestimation of the size of unmet health care needs.

## Conclusion

We recognize regional inequality and defined populations with unmet health care needs as the older male active population with basic education, the unemployed, those in the poorest income quintile group, those who usually live in thinly populated areas or in the regions of Vojvodina, Sumadija and Western Serbia, who assessed their health as poor, with chronic illnesses and functional limitations in daily activities. The responsibility of the health system in Serbia, which reflects the availability and accessibility of health care, was more dominant than the responsibilities of the respondents. Regional inequality of access to basic health services is significantly present in Serbia, and financial reasons were the most frequent in all regions. Predictors of unmet health care needs for overall reasons, which increase the likelihood of their emergence included self-perception of health status as very bad, divorced person or widower/widow, living in the Sumadija and Western Serbia region and belonging to the age groups of 27–44 and 45–64 years. The probability for those least reporting unmet health care needs were female patients, with higher education, who belong to the richest (fifth) quintile and who are unemployed.

Health policy should adopt a multidimensional approach to health care which develops incentives for the appropriate use of health services and focuses on eliminating barriers that limit accessibility and availability of health care to the entire and vulnerable population, which are the responsibility of the health system.
